# Diaphragmatic Injuries among Severely Injured Patients (ISS ≥ 16)—An Indicator of Injury Pattern and Severity of Abdominal Trauma

**DOI:** 10.3390/medicina58111596

**Published:** 2022-11-04

**Authors:** Leonhard Andreas Schurr, Claudius Thiedemann, Volker Alt, Hans Jürgen Schlitt, Markus Götz, Moritz Riedl, Stefan Martin Brunner, Daniel Popp

**Affiliations:** 1 Department of Surgery, University Medical Center Regensburg, Franz-Josef-Strauss-Allee 11, 93053 Regensburg, Germany; 2 Department of Trauma Surgery, University Medical Center Regensburg, 93053 Regensburg, Germany

**Keywords:** emergency surgery, diaphragm, injury, abdominal trauma, major trauma, laparotomy

## Abstract

*Background and Objectives:* Abdominal trauma among severely injured patients with an injury severity score (ISS) of 16 and above can lead to potentially life-threatening injuries that might need immediate surgical intervention. Traumatic injuries to the diaphragm (TID) are a challenging condition often accompanied by other injuries in the thoracoabdominal region. *Materials and Methods:* We retrospectively analyzed the occurrence and clinical course of TID among severely injured patients treated at our center between 2008 and 2019 and compared them to other groups of severely injured patients without TID. *Results:* Thirty-five patients with TID and a median ISS of 41 were treated in the period mentioned above. They were predominantly middle-aged men and mostly victims of blunt trauma as a consequence of motor vehicle accidents. A total of 70.6% had left-sided TID, and in 69.6%, the size of defect was larger than 10 cm. The diagnosis was made with computed tomography (CT) in 68.6% of the cases, while in 25.8%, it was made intraoperatively or delayed by a false-negative initial CT scan, and in 5.7%, an intraoperative diagnosis was made without preoperative CT imaging. Surgical repair was mostly conducted via laparotomy, performing a direct closure with continuous suture. A comparison to 191 patients that required laparotomy for abdominal injuries other than TID revealed significantly higher rates of concomitant injuries to several abdominal organs among patients suffering from TID. Compared to all other severely injured patients treated in the same period (*n* = 1377), patients suffering from TID had a significantly higher median ISS and a longer mean duration of hospital stay. *Conclusions:* Our findings show that TID can be seen as an indicator of particularly severe thoracoabdominal trauma that requires increased attention from the treatment team so as not to miss relevant concomitant injuries that require immediate intervention.

## 1. Introduction

Severely injured patients with an injury severity score (ISS) of 16 and above represent an interdisciplinary challenge at trauma centers worldwide. Standardized diagnostic workup followed by early clinical decision making is crucial. In clinical routine, a full-body CT scan is recommended and performed as a standard of care when potentially life-threatening injuries are suspected [1,2,3,4,5,6]. Abdominal trauma, which is often present among severely injured patients, can lead to such life-threatening conditions that need immediate surgical intervention [6,7,8]. A traumatic injury to the diaphragm (TID) is a potentially challenging condition that is often accompanied by other thoracoabdominal injuries [9,10,11]. Up to 75% of TID are caused by blunt trauma mechanisms, often motor vehicle accidents [12]. The left hemidiaphragm is affected more than twice as often as the right side. Hepatic protection and the underdiagnosis of injuries to the right side are two possible explanations for this discrepancy [12]. A male predominance of about 2.5:1–4:1 (male:female) has been reported for this injury [9,12]. The delayed clinical presentation and diagnosis of TID is possible but is becoming less likely in patients undergoing modern CT scans [11,13,14]. However, initial diagnostics have been reported to miss up to 60% of acute TIDs [15]. The overall mortality of patients with TID has been recently reported as being from 26.8 to 41% [9,11]. The literature shows no doubt about the indication for surgical treatment of TID, and the most common approach is laparotomy [10,14,16,17].

The aim of this study was to retrospectively analyze the incidence, presentation, management and outcome of adult severely injured patients with TID at our center from 2008 until 2019. Furthermore, these data were compared with those of all other severely injured patients in the same period regarding their presentation and general clinical course. A more specific comparison regarding abdominal injury patterns was performed using data from severely injured patients with relevant injuries other than TID that required laparotomy.

## 2. Patients and Methods

All severely injured patients treated at our medical center (a level-1 trauma center) between 2008 and 2019 were screened for this retrospective analysis. Inclusion criteria were ISS ≥ 16 and age ≥ 18 years.

Correspondingly, exclusion criteria were defined as ISS < 16 and age < 18 years. Ethical approval was obtained by the Ethics Committee of the University of Regensburg (21-2260-104) on 24 March 2021. Data collection was primarily conducted by study assistants (24 h, 7 d a week) independently from the treatment algorithm as part of a prospective institutional trauma database. More specific data were filled in by the authors retrospectively. All included patients were primarily evaluated with regard to the occurrence of TID and other relevant abdominal injuries, as well as the necessity of laparotomy. The patients were then divided into three groups:

*Group 1:* Severely injured patients with TID (*n* = 35).
*Group 2:* Severely injured patients without TID, but with other relevant abdominal injuries requiring laparotomy (*n* = 191).
*Group 3:* All other severely injured patients without TID (*n* = 1377).

Data collection included demographics, diagnostics, ISS, specific abdominal injury patterns, management, clinical course (including duration of ventilation, length of stay on ICU and overall hospitalization) and short-term outcome (30-day mortality). Detailed information regarding the specifics of TID and its operative management was obtained.

Group 1 was thoroughly analyzed in a descriptive fashion. Furthermore, we compared the groups regarding possible differences in the parameters mentioned above. Beyond that, the eventual prognostic relevance of TID was investigated. Therefore, we analyzed possible differences in the specific abdominal injury patterns among groups 1 and 2.

Statistical analysis was performed using SPSS version 25 software (SPSS Inc., Chicago IL, USA). A univariate data analysis was performed to compare the three groups. The Chi-Square-Test (x2 Test) and Fisher’s exact test were used to analyze binary or nominal target variables. Normal distribution was examined using the Kolmogorov–Smirnov test. Unpaired samples t-test was used to compare mean values among the groups. Median values were compared by performing the Mann–Whitney-U-test. *P*-values of 0.05 or lower were considered statistically significant.

## 3. Results

During the period defined above, 1603 severely injured patients (ISS ≥ 16) were treated at our center, 35 of which were diagnosed with and treated for TID (group 1). This results in an 2.18% incidence of TID among all severely injured patients. A total of 191 patients requiring urgent laparotomy for abdominal injuries other than TID (group 2) as well as 1377 severely injured patients without TID (group 3) were admitted in the same time frame.

The table below (Table 1) contains demographic data and median ISS values among the three groups.

The comparison of the median age and sex ratio among the three groups showed no statistically significant differences. A significantly higher median ISS was present in group 1 (41) compared to group 3 (27, *p* < 0.001). Group 2 showed a lower median ISS (34) compared to group 1, but without statistical significance (*p* = 0.16).

In Table 2, the parameters of initial management and clinical course among the three groups are displayed.

Patients in group 1 required preclinical cardiopulmonary resuscitation (CPR) more often (14.8%) than groups 2 (8.6%) and 3 (7.1%), yet without statistical significance. The rate of preclinical chest tube insertions was significantly higher in group 1 (37.0%) compared to groups 2 (19.2%, *p* = 0.039) and 3 (11.6%, *p* < 0.001). Patients in group 1 had a mean length of stay on the intensive care unit (ICU) of 14.4 days, which showed a tendency to be longer compared to group 3 (10.6 d) without statistical significance (*p* = 0.065). Group 2 had a similar length of stay in the ICU (14.8 d) compared to group 1. The duration of invasive ventilation was not significantly different between groups 1 (7.7 d), 2 (8.1 d) and 3 (6.1 d) either. However, the overall length of stay in the hospital showed a significant difference (*p* = 0.024) between groups 1 (26.6 d) and 3 (19.6 d). Patients in group 2 were hospitalized slightly more briefly (24.9 d) compared to group 1, without statistical significance. The 30-day mortality in group 1 was 20%, compared to 18.4% in group 2 and 17.5% in group 3, without a significant difference.

In group 1, 27 patients (77.1%) were treated primarily at our medical center, while 8 (22.9%) were transferred after external primary care. The mechanism of injury was blunt trauma in 29 (82.9%), penetrating trauma in 3 (8.6%) and a combination in the other 3 (8.6%) cases. Most of the patients suffered from motor vehicle accidents. Thirty-one patients (88.6%) had accompanying thoracic injuries such as rib fractures, lung contusions or (hemato)pneumothoraces.

In 24 patients (70.6%), TID was documented in the left hemidiaphragm, while 9 (26.5%) had right-sided injuries, 1 (2.9%) had a bilateral injury and 1 was not specified. The size of defect was measured at <5 cm in 5 (21.7%), 5–10 cm in 2 (8.7%) and >10 cm in 16 (69.6%) cases, and in 12 patients, no measurements were taken or documented. The thoracic herniation of abdominal organs was documented in 16 patients (45.8%).

TID was diagnosed using CT in 24 (68.6%) patients. The diagnosis was made intraoperatively in six (17.1%) and delayed in three (8.6%) of the present cases, despite initial CT imaging. Two patients (5.7%) required emergency operation in which TID was found, without a preoperative CT scan. Of 33 operated patients, 31 (93.9%) underwent preoperative CT imaging and 2 (6.1%) underwent surgery directly. Two patients died without surgical intervention. In 24 of 33 operated cases (72.7%), immediate surgical repair was carried out. Two patients (6.1%) were operated on within 12 and between 12 and 48 h after their accident, respectively, whilst five (15.2%) did not undergo surgery until more than 48 h after trauma. One patient died during his emergency laparotomy. Another patient was diagnosed with a symptomatic diaphragmatic hernia, presumably associated with prior severe trauma, 6 months after the accident.

The repair of TID was performed via laparotomy in 29 of all operated cases (87.9%), via thoracotomy in 2 (6.1%) and via a combined approach in 2 (6.1%). Thirty (96.8%) patients received direct repair; in one (3.2%), mesh augmentation was performed. In four cases, the repair was not specified. Direct suture was documented using interrupted stiches in 6 (27.3%) and continuous suture in 16 (72.7%) cases. In 11 patients, the surgical technique was not specified. Among the 31 cases who underwent laparotomy, 14 (45.2%) required just one operation, 12 (38.7%) underwent a second look, 2 (6.5%) patients underwent three and four operations, respectively, and 1 patient (3.2%) had six and eight overall operations, respectively. Reoperations were primarily necessary due to concomitant abdominal injuries, such as hollow organ perforations or solid organ lacerations, not because of TID itself.

The exact abdominal injury patterns were analyzed and compared between groups 1 and 2. The following table (Table 3) shows the numbers of patients with injuries to specific abdominal organs and the corresponding *p*-values after statistical comparison.

The results show that injuries to the stomach, large bowel, liver, pancreas, kidneys and/or adrenal glands, mesentery and aorta occurred significantly more often among patients in group 1 compared to group 2. No statistical significance was present regarding injuries to the duodenum, small bowel, spleen and vena cava. Injuries to the small intestine appeared to occur more often in group 2. All other organs mentioned above were harmed more often among patients in group 1.

## 4. Discussion

TID is a rare entity that usually occurs as part of a complex injury pattern among severely injured patients. This is consistent with previous findings in the literature [18]. In this study, we investigated the characteristics of this injury. Furthermore, to evaluate the relevance of TID among severely injured patients, we compared our collective of patients with TID (group 1) to patients with other relevant abdominal injuries requiring laparotomy (group 2) as well as all other severely injured patients without TID (group 3).

Our three collectives showed no relevant difference in age or sex ratio.

The median ISS of patients in group 1 was significantly higher compared to group 3 and slightly higher, but without statistical significance, compared to group 2. Our study specifically concentrates on severely injured patients with an ISS ≥ 16, so opportunities for comparison with previous publications regarding median or mean ISS are limited. A median ISS of 41 in the analyzed group of TID patients indicates an above-average severity of trauma in spite of our preliminary patient selection. One previous study has reported a similar mean ISS of 41.33, but specifically among their fatalities with TID, compared to a mean ISS of 17.67 among survivors [17]. In another article, almost 95% of patients with blunt TIDs were severely injured, with an ISS of ≥ 16 [9].

In the literature, both blunt and penetrating injury mechanisms have been reported as causing TID. In our collective, TID was diagnosed after blunt trauma in 29 cases (82.9%), almost equal to a study performed in a Greek trauma center, which reported an 80% rate of injuries following blunt trauma [17]. Contrary data were published in an American analysis with a 67% rate of penetrating traumas, of which two thirds were caused by gunshot wounds and another third by stab wounds [19]. An article from Turkey reported a similar rate of 68% penetrating mechanisms [10]. The latter discussed demography and regional “sociological conditions” as a possible explanation for higher rates of penetrating injuries.

We found a high rate (88.6%) of accompanying thoracic injuries in patients with TID, which corresponds with the significantly higher rate of preclinical chest tube insertions among patients in group 1 and underlines overall injury severity in patients with TID. A similarly high rate of 90% associated thoracic injuries has been reported in a recent review [11]. Further studies need to be conducted regarding specific patterns of thoracic injuries accompanying TID.

The predominance of left-sided TID reported in the literature [10,12,16,17] was present in our collective of patients as well. In the past, various possible explanations have been reported, including higher strength of the right hemidiaphragm, protection by the liver, the underdiagnosis of right-sided injuries and the vulnerability of the left hemidiaphragm in embryonic fusion zones [12].

Diagnosis was made mostly by CT imaging, which nowadays is the diagnostic standard in severely injured patients with potentially life-threatening thoracoabdominal injuries [2]. Recently, the higher sensitivity and specificity of CT imaging over conventional chest X-ray in detecting penetrating TIDs has been described [20]. The possibility of the delayed presentation of TID is well known [13]. In our collective, one patient presented with a symptomatic diaphragmatic hernia months after blunt trauma. Eight cases of TID were diagnosed intraoperatively. If those patients had not been operated on due to other concomitant injuries, their TID would have been missed as well in the acute phase. Overlooking TID in severely injured patients has been reported to happen frequently, leading to a rise in mortality [21]. Therefore, we suggest always considering the presence of TID in cases of unclear complaints regarding the upper abdominal area following blunt trauma. In cases of penetrating thoracoabdominal trauma, diagnostic laparoscopy should be considered liberally to look for TID [20,22].

In most of our cases, immediate surgical repair was performed via laparotomy, using continuous suture without mesh augmentation. Two patients without concomitant abdominal injuries underwent repair by thoracotomy, and two needed a combined approach. Laparotomy has been reported as the most commonly used approach [10,16,17]. We agree with this approach, especially because of the high rate of concomitant abdominal injuries that can accompany TID and that sometimes might not be seen in their full extent on CT scans. Their relevance could be missed when performing a primary thoracotomy. If an isolated TID following penetrating trauma is found, laparoscopic repair is feasible and seems to be superior to open repair regarding the length of hospital stay [23]. In our opinion, an open surgical approach should still be preferred in severely injured patients after blunt trauma, due to the high rate of intraabdominal concomitant injuries in our patient collective. In the presence of severe thoracic injuries, a combined abdominal and thoracic approach can be appropriate.

As already mentioned, we performed a detailed comparison of abdominal injury patterns between our collective of patients with TID (group 1) and patients with other relevant injuries to abdominal organs undergoing laparotomy (group 2). Our results showed that patients suffering from TID had significantly higher rates of injuries to the stomach, liver, pancreas, mesentery, large bowel, kidneys and/or adrenal glands and aorta. Interestingly, injuries to the small intestine tended to occur more often in group 2, whilst all other organs were injured more often in group 1. These findings show that TID can be seen as an indicator of relevant concomitant injuries to several other abdominal organs. No previous studies have evaluated the prognostic relevance of TID regarding the presence of other abdominal injuries by performing a similar comparison. As a consequence, in clinical practice, we suggest that the finding of TID in a severely injured patient should always be met with increased attention from the medical team, so as not to miss other relevant injuries, especially to abdominal organs, that might need immediate interventions.

The length of stay on the ICU, duration of invasive ventilation and 30-day mortality showed no significant differences among our three groups. However, the overall duration of hospital stay was significantly longer in group 1 compared to group 3, which again can be seen as an indicator for the exceptional severity of trauma, necessitating a prolonged recovery time.

Limitations of this study include its design as a retrospective single-center analysis and the relatively small number of patients included in group 1, which, however, is due to the rare occurrence of TID. Further investigations need to be conducted to confirm the prognostic relevance of TID and to investigate independent risk factors for the presence of TID itself.

## 5. Conclusions

TID is a rare injury, even among severely injured patients, which mostly occurs on the left side among middle-aged men following blunt trauma, often caused by motor vehicle accidents. CT imaging is the diagnostic method of choice, and the surgical repair of TID is always indicated. Laparotomy should be the approach of choice in severely injured patients, and direct suture is feasible in almost all cases. TID is an indicator of severe abdominal injury patterns with significantly higher rates of concomitant injuries to several abdominal organs compared to severely injured patients undergoing laparotomy for injuries other than TID. An exceptional overall severity of trauma with prolonged hospitalization is associated with the presence of TID. Therefore, the finding of TID should always be met with increased attention to the search for other relevant abdominal injuries, as well as quick therapeutic decision making.

## Figures and Tables

**Table 1:** Demographic characteristics and ISS.

	Group 1 (*n* = 35)	Group 2 (*n* = 191)	Group 3 (*n* = 1377)	*p*-Value (1 vs. 2)	*p*-Value (1 vs. 3)
Median age	43 a (18–85)	42 a (18–92)	48 (18–94)	0.84	0.37
Sex ratio M:F	23:12 (M: 65.7%)	124:67 (M: 64.9%)	1024:353 (M: 74.4%)	0.93	0.25
Median ISS	41 (17–75)	34 (16–75)	27 (16–75)	0.16	**<0.001**

**Table 2:** Initial treatment and clinical course.

	Group 1 (*n* = 35)	Group 2 (*n* = 191)	Group 3 (*n* = 1377)	*p*-Value (1 vs. 2)	*p*-Value (1 vs. 3)
Prehospital CPR	14.8%	8.6%	7.1%	0.31	0.12
Prehospital chest tube insertions	37.0%	19.2%	11.6%	**0.039**	**<0.001**
Mean length of stay on ICU	14.4 d (SD = 11.7)	14.8 d (SD = 15.1)	10.6 d (SD = 12.0)	0.88	0.065
Mean duration of invasive ventilation	7.7 d (SD = 8.3)	8.1 d (SD = 9.1)	6.1 d (SD = 8.5)	0.84	0.28
Mean overall length of stay	26.6 d (SD = 17.7)	24.9 d (SD = 22.2)	19.6 d (SD = 16.7)	0.69	**0.024**
30-day mortality	20%	18.4%	17.5%	0.83	0.73

**Table 3:** Abdominal injury patterns among groups 1 and 2.

Injured Organ:	Group 1 (*n* = 35)	Group 2 (*n* = 191)	*p*-Value
Stomach	3 (8.6%)	3 (1.6%)	**0.049**
Duodenum	2 (5.7%)	1 (0.5%)	0.063
Small bowel	3 (8.6%)	20 (10.5%)	1
Large bowel	8 (22.9%)	15 (7.9%)	**0.007**
Liver	17 (48.6%)	48 (25.1%)	**0.005**
Spleen	16 (45.7%)	67 (35.1%)	0.23
Pancreas	7 (20%)	5 (2.6%)	**<0.001**
Kidneys a/o adrenal glands	8 (22.9%)	13 (6.8%)	**0.003**
Mesentery	11 (31.4%)	15 (7.9%)	**<0.001**
Aorta	5 (14.3%)	7 (3.7%)	**0.01**
V. cava	1 (2.9%)	0 (0%)	0.16

## Data Availability

The datasets used in this investigation can be requested from the corresponding author.
